# TLR agonists enhance responsiveness of inflammatory innate immune cells in HLA-B*57-positive HIV patients

**DOI:** 10.1007/s00109-020-01996-7

**Published:** 2020-12-05

**Authors:** L. Dold, L. Zimmer, C. Schwarze-Zander, C. Boesecke, R. Mohr, J.-C. Wasmuth, K. Ommer, B. Gathof, B. Krämer, J. Nattermann, C. P. Strassburg, J. K. Rockstroh, U. Spengler, B. Langhans

**Affiliations:** 1 Department of Internal Medicine I, University Hospital of Bonn (UKB), Venusberg-Campus 1, 53127 Bonn, Germany; 2 German Center for Infection Research (DZIF), Partner Site Cologne-Bonn, Bonn, Germany; 3 Institute of Transfusion Medicine, University Hospital of Cologne, Cologne, Germany

**Keywords:** HIV, HLA-B*57, cART, Bacterial infections, Toll-like receptor stimulation, Inflammatory immune response

## Abstract

**Abstract:**

HLA-B*57 affects the course of HIV infection. Under antiretroviral therapy, its effects cannot be explained by outstandingly efficient T cell responses alone but may also involve cells of innate immunity. Studying *in vitro* stimulation with Pam3CSK4, *E. coli* LPS-B5 and CpG-ODN-2216, we observed greater induction of IL-6/IL-1beta double-positive CD14^+^CD16^++^ monocytes as well as IFN-gamma-positive cytotoxic CD56^high^CD16^neg^ NK cells in HLA-B*57- versus HLA-B*44-positive HIV patients, while TNF-alpha induction remained unchanged. Differences were not seen in the other monocyte and NK cell subsets or in HLA-matched healthy controls. Our findings show that, in virally suppressed HIV infection, HLA-B*57 is associated with enhanced responsiveness of inflammatory innate immune cells to TLR ligands, possibly contributing to increased vulnerability in sepsis.

**Key messages:**

• HLA-B*57 is a host factor affecting clinical outcomes of HIV infection.

• HLA-B*57 modifies inflammatory subsets of NK cells and monocytes in HIV infection.

• In HLA-B*57-positive HIV patients TLR agonists induce enhanced IL-6/IL-1beta in monocytes.

• NK cells from HLA-B*57 HIV patients release more IFN-gamma upon TLR costimulation.

• HLA-B*57 is linked to enhanced inflammatory responsiveness to TLR ligands.

**Supplementary Information:**

The online version contains supplementary material available at 10.1007/s00109-020-01996-7.

## Introduction

The course of human immunodeficiency virus (HIV) infection is highly variably owing to complex interactions between the virus and the host. Among multiple factors HLA class I loci have been identified as pivotal host factors affecting clinical outcomes. In particular, HLA-B*57 appears to be linked with low level viremia and delayed disease progression, which has been attributed to an exceptional efficacy of HLA-B*57-restricted T cells to control HIV infection [1]. While this protective effect was consistently observed in untreated HIV patients, even across different HLA-B*57 subtypes and diverse HIV strains [2], the risk to die from bacterial infection and sepsis was unexpectedly found to be substantially increased in HLA-B*57-positive patients, whose HIV replication was long-term suppressed by antiretroviral therapy [3]. This observation is not explained by the outstandingly efficient T cell responses associated with HLA-B*57 but may be a hint that the HLA system can also modify innate immune responses under certain conditions.

Sepsis is now considered to reflect a dysregulated systemic inflammatory immune response to pathogen-associated molecular patterns (PAMPs) and damage-associated molecular patterns (DAMPs), which trigger a variety of different receptors such as Toll-like receptors (TLRs) primarily on cells of the innate immune system, e.g. monocytes and natural killer (NK) cells [4]. This induces an abundant release of pro-inflammatory cytokines, which mediates multiple deleterious effects and causes organ dysfunctions that can lead to death ultimately.

Monocytes rapidly respond to bacterial infections and produce multiple inflammatory cytokines. Monocytes are dysregulated in HIV infection and can be classified into several subtypes. In particular, non-classical CD14^+^CD16^++^ monocytes are expanded in bacterial infections as a potent source of cytokines [5].

Unlike T lymphocytes, which upregulate TLRs only upon immune activation [6], NK cells express TLRs constitutively, and thus, can be activated directly by bacteria. They exert cytotoxicity against cells and are a further source of inflammatory and immunoregulatory cytokines such as TNF-alpha and IFN-gamma [7].

Since it is not readily explained why HLA-B*57-positive individuals with controlled HIV infection exhibit increased susceptibility to die from bacterial infections, we studied in vitro effects of TLR stimulation on monocytes and NK cells isolated from treated HIV patients carrying the HLA-B*57 type versus the HLA-B*44 control type and compared them to healthy controls with the same HLA types.

## Material and methods

### Patients

Between June 2018 and November 2019, we recruited HLA-B*57 and HLA-B*44 HIV-mono-infected patients from the HLA-typed prospective Bonn cohort who had persistently suppressed HIV replication on antiretroviral therapy and consented to participate [3]. Control blood samples were obtained from HLA-matched healthy volunteers via the Cologne University blood banking service. All participants gave their written informed consent to participate in this study which had been approved by the ethics committee of Bonn (decision 119/18) and Cologne (decision 18-234) University.

### Reagents

Triacylated lipoprotein Pam3CSK4 (TLR1/2 ligand; 1 mg/ml), ultrapure preparation of lipopolysaccharide from *E. coli* LPS-B5 Ultrapure (TLR4 ligand; 1 mg/ml), and CpG-ODN-2216 (Class A CpG oligonucleotide; TLR9 ligand; 1 mg/ml dissolved in Lipofectamine2000® transfection reagent (Invitrogen, Darmstadt, Germany) to ensure easy access to endosomes) were purchased as synthetic TLR ligands from InvivoGen (Toulouse, France).

### Cell preparations from peripheral blood

Peripheral blood mononuclear cells (PBMC) were isolated by Ficoll-Paque density gradient centrifugation (PAA Laboratories, Cölbe, Germany) from heparinized blood and cryopreserved in liquid nitrogen until analysis. Monocytes were studied directly in thawed PBMC, while NK cells and CD3^+^ T cells were further separated immunomagnetically using MACS cell separation kits (NK Cells Isolation Kit, Pan T Cell Isolation Kit, both Miltenyi Biotec, Bergisch Gladbach, Germany). To ensure NK and T cell purity of > 95% (controlled via FACS), we removed monocytes by plating cells for additional 2 h.

### Functional analysis TLR-stimulated cells

#### Monocytes

TLR activation of monocytes was analysed concerning induction IL-1beta, IL-6 and TNF-alpha. Thawed PBMC were incubated in RPMI1640 with and without Pam3CSK4, LPS-B5 and CpG-ODN-2216, respectively. After 4 h, brefeldin A (BFA, 0.5 μg/ml; Enzo Life Sciences GmbH, Lörrach, Germany) was added for additional 16 h. Then, dead and viable cells were discriminated by Zombie Aqua^TM^ staining (BioLegend, London, UK). After adding TruStain FcX® (BioLegend), cells were stained with anti-CD3 (APC-Cy7-labelled), anti-CD16 (FITC-labelled) and anti-CD14 (PerCP-labelled) (all BioLegend). Then, cells were fixed and permeabilized (Cytofix/Cytoperm Kit; BD Pharmingen) to enable intracellular staining with PE-labelled anti-IL-1beta (Life Technologies GmbH, Frankfurt, Germany), BV421-labelled anti-IL-6 and PE-Cy7-labelled anti-TNF-alpha (both BioLegend). Finally, samples were analysed on a FACSCanto II (BD Biosciences, Heidelberg, Germany) with the FlowJo V10 software (TreeStar Inc., Ashland, OR, USA). Percent IL-1beta-, IL-6- and TNF-alpha-positive cells measured in the classical (CD14^++^CD16^–^), intermediate (CD14^++^CD16^+^) and non-classical (CD14^+^CD16^++^) monocyte subsets (Supplementary Figure 1). Fluorescence minus one (FMO) controls and isotype controls were performed for all antibody panels to define positive signals and confirm proper compensation.

#### NK cells

TLR activation of NK cells was analysed concerning induction IFN-gamma and TNF-alpha as well as CD107a degranulation in response to K562 target cells. Purified NK cells were pre-activated overnight with IL-2 and then cultured with and without Pam3CSK4, LPS-B5 and CpG-ODN-2216, respectively. After 16 h, NK cells were divided into either co-culture with K562 effector cells (effector to target ratio of 1:2) or kept in culture with medium alone. Next, FITC-conjugated CD107a (BD Biosciences, Heidelberg, Germany) was added. After 1 h, Golgi Stop (BD Biosciences) and BFA (Enzo Life Sciences GmbH) were added for additional 3 h. Then, cells were stained with Zombie AquaTM (BioLegend, London, UK) followed by staining with anti-CD3 (APC-Cy7-labelled), anti-CD56 (Brilliant Violet 421-labelled) and anti-CD16 (PerCP-labelled). After fixation and permeabilization, cells were stained intracellularly with anti-IFN-gamma (PE-labelled) and anti-TNF-alpha (PE-Cy7-labelled) (all BioLegend). Percent IFN-gamma-, TNF-alpha- and CD107a-positive CD56^dim^CD16^pos^, CD56^dim^CD16^neg^ and CD56^high^CD16^neg^ NK cells were measured before and after TLR stimulation as well as after co-culture of NK cells with K562 target cells in the presence and absence of TLR stimulation (Supplementary Figure 2).

#### CD8^+^ and CD4^+^ T cells

Analogous to NK cells, IFN-gamma-, TNF-alpha- and CD107a induction by TLR ligands was measured flow cytometrically concerning activation of CD3^+^CD8^+^ and CD3^+^CD4^+^ T cells. In addition, we studied lectin-dependent cellular cytotoxicity (LDCC) assays against Con-A-loaded P815 cells [8].

### Statistical analysis

Differences were compared by the Mann-Whitney *U* test, Wilcoxon matched-pairs signed-rank test and Student’s *t* test as appropriate. Multiple comparisons were done by one-way ANOVA with Bonferroni correction. Calculations were performed with the SPSS statistics software (version 24) and GraphPad Prism 8.0 software packages (GraphPad Prism, San Diego California, USA), respectively.

## Results

### Clinical features of HIV patients and healthy controls

We recruited each six HLA-B*57-positive and six HLA-B*44-positive HIV-mono-infected patients, whose HIV RNA levels were below detection on antiretroviral therapy. Patients did not have evidence of liver disease; also clinical data including CD4^+^, CD8^+^ and NK cell counts, and antiretroviral therapy did not differ between the groups (Table 1). We matched the HIV patients to 12 healthy controls (6 HLA-B*57 and HLA-B*44-positive each) with respect to sex and age.

**Table 1:** Characteristics of HIV-infected patients and healthy controls

a) Analysis of HIV-infected patients												
Patient-Id	#1	#2	#3	#4	#5	#6	#7	#8	#9	#10	#11	#12
HLA-B type	B*57	B*57	B*57	B*57	B*57	B*57	B*44	B*44	B*44	B*44	B*44	B*44
Age (years)	57	58	43	60	42	59	54	49	80	52	54	71
Sex (male,female)	Male	Male	Male	Male	Female	Male	Male	Male	Female	Male	Male	Male
Clinical data												
ALT (U/L)	39	22	48	18	17	55	48	25	30	23	13	62
AST (U/L)	31	24	19	28	19	39	34	40	28	28	17	41
HIV status												
CD4 count	934	478	533	475	947	717	618	501	291	314	630	597
CD8 count	1031	750	572	294	1307	1260	897	855	1361	718	1586	1157
NK cell count	169	119	314	161	224	308	168	235	457	188	315	199
HIV RNA(IU/mL)	< 40	< 40	< 40	< 40	< 40	< 40	< 40	< 40	< 40	< 40	< 40	< 40
Liver stiffness(kPa)	n.d.	6.1	4.6	n.d.	4.4	n.d.	6.1	6.1	n.d.	3.8	4.8	5.8
HCV status	Negative	Negative	Negative	Negative	Negative	Negative	Negative	Negative	Negative	Negative	Negative	Negative
HIV therapy	FTC, TDF,DTG	FTC, TDF,RLP	FTC, TDF,RLP	FTC, TDF,DTG	FTC, TDF,RLP	DRV, RAL,RTV	FTC, TDF,DTG	FTC, TDF,DTG	DRV, RAL,RTV	DRV, RAL,RTV	FTC, TDF,DRV, CC	TDF, DTG,DRV, RTV
b) Analysis of healthy controls												
Control-Id	#1	#2	#3	#4	#5	#6	#7	#8	#9	#10	#11	#12
HLA-B type	B*57	B*57	B*57	B*57	B*57	B*57	B*44	B*44	B*44	B*44	B*44	B*44
Age (years)	58	24	28	26	62	58	51	77	42	25	52	42
Sex (male,female)	Female	Female	Male	Male	Male	Male	Male	Female	Male	Male	Male	Male

* Emtircitabine = FTC, Tenofovir = TDF, Dolutegravir = DTG, Rilpivirin = RLP, Darunavir = DRV, Raltegravir = RAL, Ritonavir = RTV (Booster), Cobicistat = CC (Booster)

### TLR ligands do not modify CD8^+^ and CD4^+^ cells and LDCC in HIV-positive patients

**Fig. 1:**
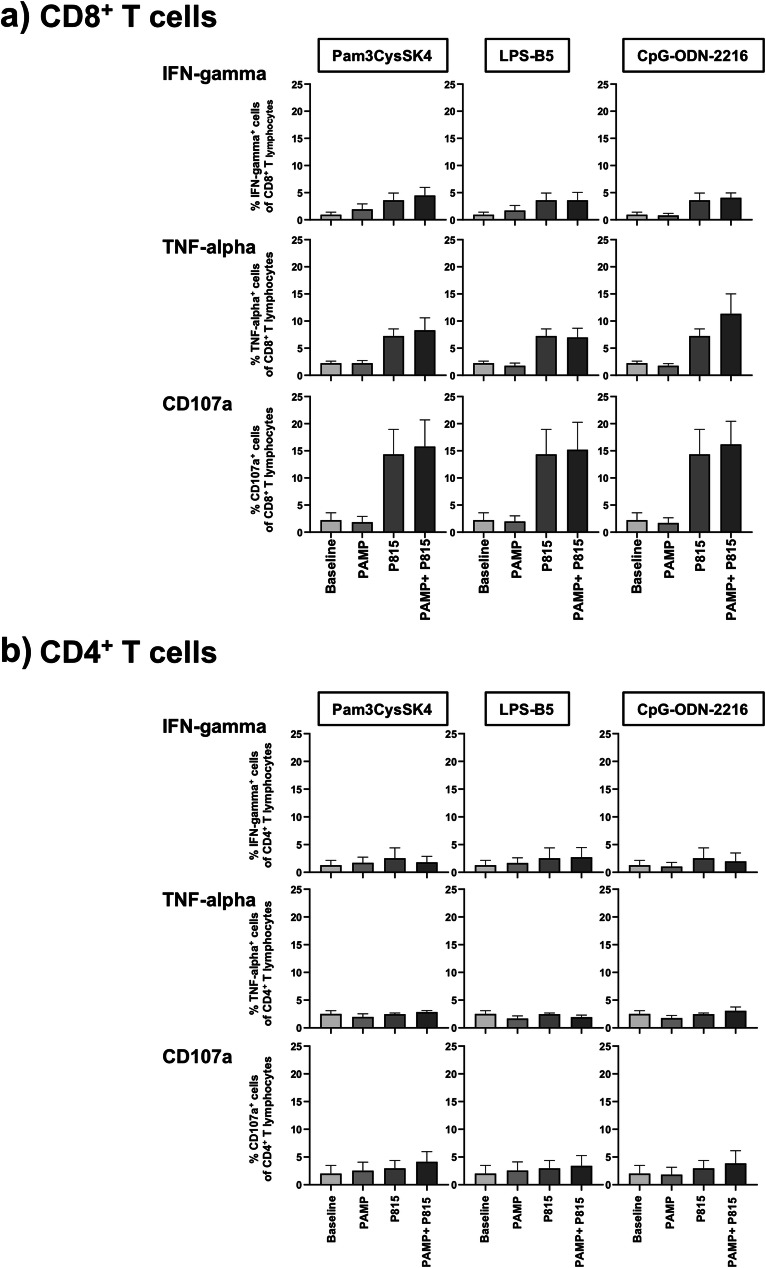
Frequencies of IFN-gamma-, TNF-alpha and CD107a-positive T lymphocytes. These figures illustrate that stimulations with Pam3CysSK4, LPS-B5 and CpG-ODN-2216 did not modify cytokine production (IFN-gamma, TNF-alpha) and CD107a degranulation in CD8^+^ (**a**) and CD4^+^ T cells (**b**) and did not affect P815-induced LDCC responses in HIV-positive patients.

Since T cells can modify responsiveness of monocytes and NK cells, we checked T cell activation upon TLR stimulation in HIV patients. However, our TLR ligands did not affect cytokine production, CD107a degranulation and LDCC in CD8^+^ or CD4^+^ T cells (Fig. 1).

### TLR stimulation enhances activity of non-classical CD14^+^CD16^++^ monocytes in HLA-B*57-positive HIV patients

Since cytokines released by monocytes pivotally determine survival during bacterial infections, we analysed induction of IL-6, IL-1beta and TNF-alpha separately in TLR-stimulated classical CD14^++^CD16^−^, intermediate CD14^++^CD16^+^ and non-classical CD14^+^CD16^++^ monocytes.

**Fig. 2:**
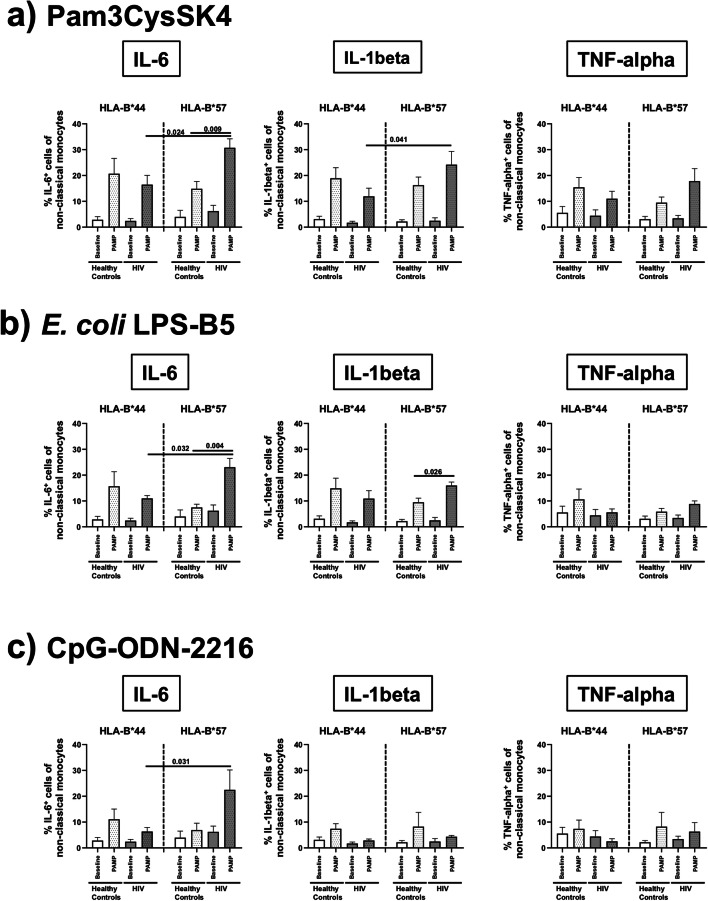
Frequencies of IL-6-, IL-1beta- and TNF-alpha-positive non-classical CD14^+^CD16^++^ monocytes. This figure illustrates increased frequencies of IL-6-positive and IL-1beta-positive cells in the non-classical CD14^+^CD16^++^ monocyte subsets of HLA-B*57-positive HIV patients after exposure to Pam3CSK4 (**a**), LPS-B5 (**b**) and CpG-ODN-2216 (**c**) compared to the control HIV patients with HLA-B*44 and healthy controls with HLA-B*57. Stimulation with any TLR ligand did not differentially alter TNF-alpha. *P* values refer to significances obtained by unpaired non-parametric Mann-Whitney *U* test for the differences marked by bars

**Fig. 3:**
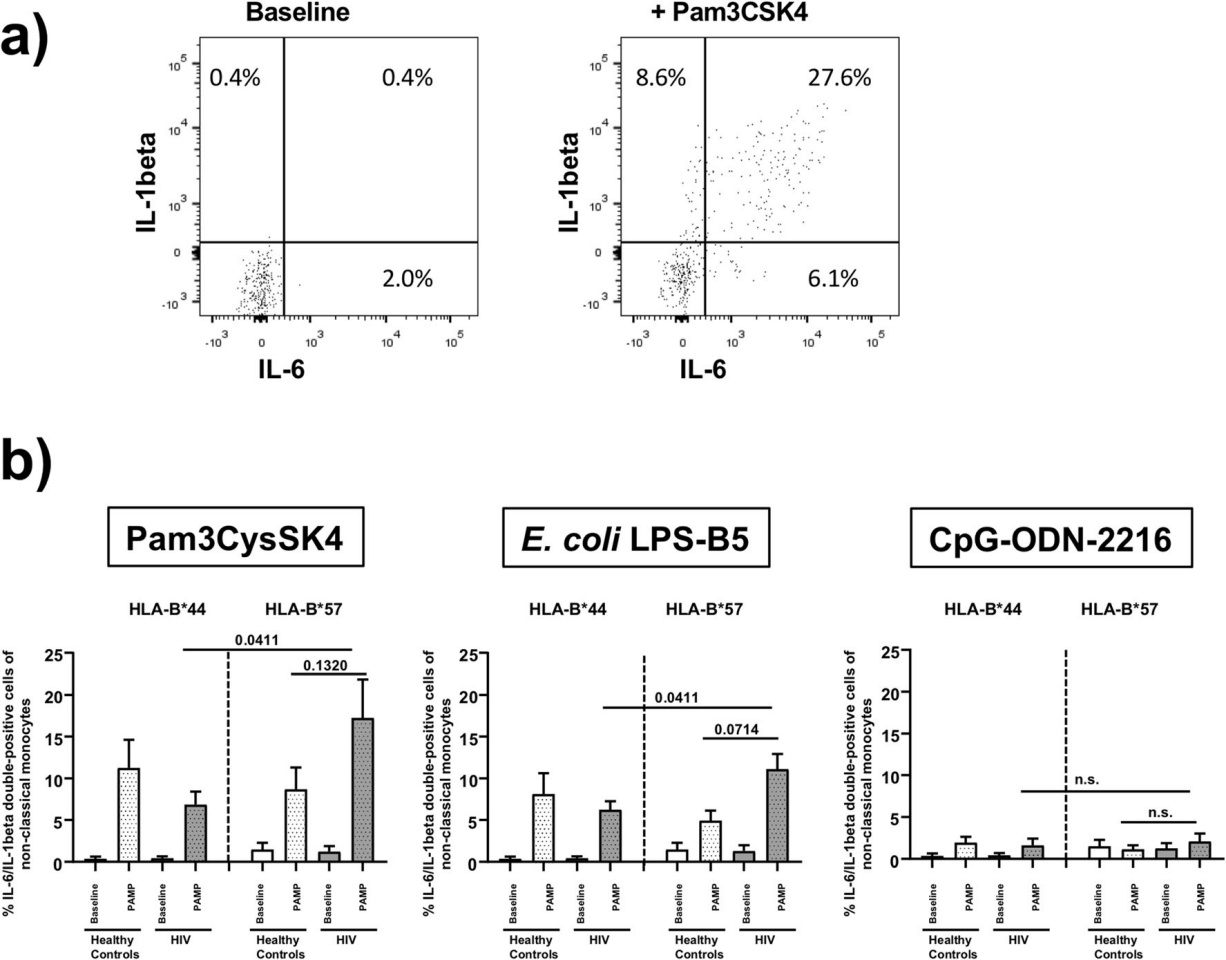
Frequencies of IL6/IL-1beta-double-positive cells of non-classical CD14^+^CD16^++^ monocytes. Panel **a** illustrates representative dot plots of IL-6/IL-1beta-double-positive non-classical monocytes at baseline versus upon stimulation with Pam_3_CysSK4 in HLA-B*57-positive patient #2. Panel **b** summarises the frequencies of IL-6/IL-1beta-double-positive CD14^+^CD16^++^ non-classical monocytes in HLA-B*57-positive HIV patients versus patients with HLA-B*44 and healthy controls. *P* values refer to significances obtained by unpaired non-parametric Mann-Whitney *U* test for the differences marked by bars

While none of the TLR ligands differentially modified monocyte responses between HLA-B*57- and HLA-B*44- positive healthy controls nor were any differential responses noted in the classical and intermediate monocyte subsets of the HIV patients, we observed greater frequencies of IL-6-positive and to some lesser extent also IL-1beta-positive cells in the non-classical CD14^+^CD16^++^ monocyte subset of HLA-B*57-positive HIV patients after exposure to Pam3CSK4, LPS-B5 and CpG-ODN-2216 than in the HIV patients with HLA-B*44 (Fig. 2). In contrast, TNF-alpha-positive non-classical monocytes did not show such a difference. Moreover, frequencies of IL-6-positive non-classical monocytes were significantly higher after Pam3CSK4- and LPS-B5 stimulation in HLA-B*57-positive HIV patients than in HLA-B*57-positive healthy controls. Finally, further flow cytometric analysis revealed that the IL-6/IL-1beta double-positive subset of CD14^+^CD16^++^ non-classical monocytes was the major source of pro-inflammatory cytokines in HLA-B*57-positive HIV patients after TLR stimulation (Fig. 3).

### Activation of TLR-stimulated CD56^high^CD16^neg^ NK cells is enhanced in HLA-B*57-positive patients with HIV infection

**Fig. 4:**
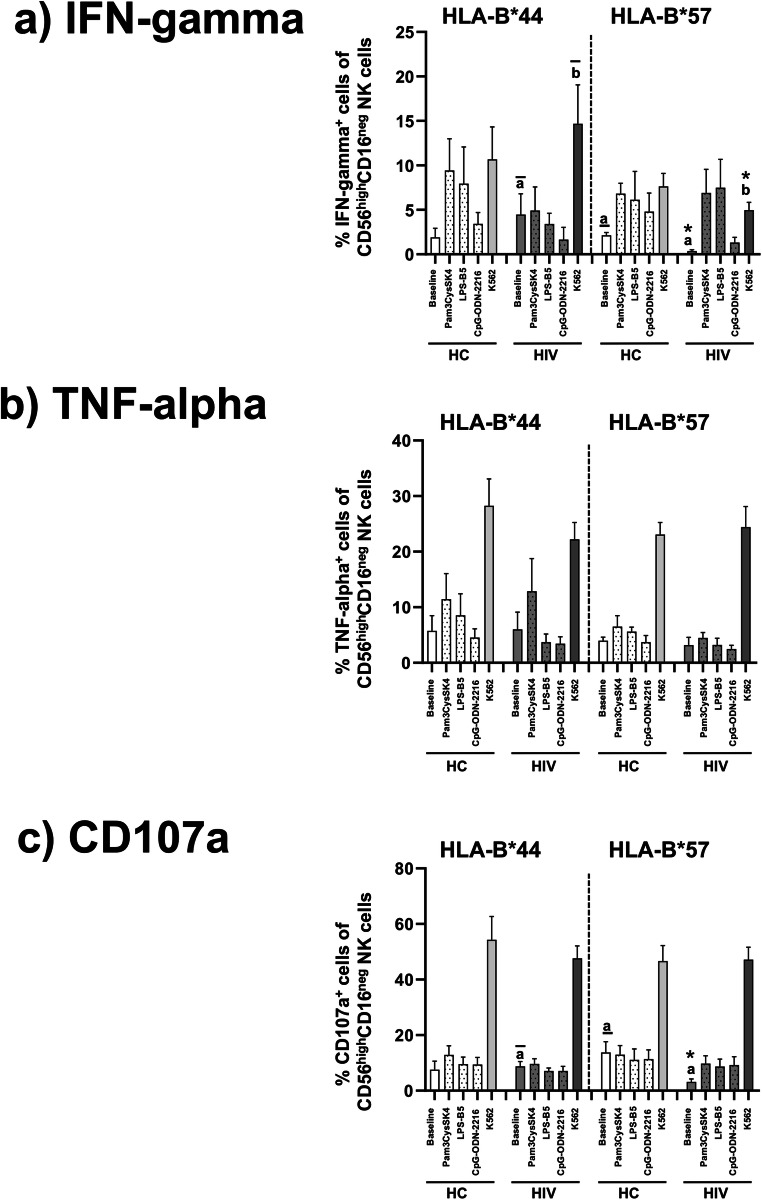
Frequencies of IFN-gamma-, TNF-alpha- and CD107a-positive CD56^high^CD16^neg^ NK cells. This figure demonstrates reduced frequencies of IFN-gamma-positive (**a**) and CD107a-positive cells (**c**) at baseline as well as in response to K562 target cells in the CD56^high^CD16^neg^ NK cell subset of HLA-B*57-positive HIV patients compared to HIV patients with HLA-B*44, whereas similar differences were not observed for TNF-alpha (**b**). *P* values refer to significances obtained by non-parametric Wilcoxon-matched pairs signed rank test and unpaired Mann-Whitney *U* test for the differences marked by letters. Error bars represent SEM and asterisks indicate significances *p* < 0.05 in pairwise statistical comparisons: In panels a and c, the letters “a*” refer to the comparisons between baseline data in the HIV patients with HLA-B*57 versus baseline data in HIV patients with HLA-B*44 ($\overline{a}$) and healthy controls with HLA-B*57 (a), respectively. Letters “b*” refer to K562-stimulation in HIV patients with HLA-B*57 versus K562-stimulation in HIV patients with HLA-B*44 ($\overline{b}$)

**Fig. 5:**
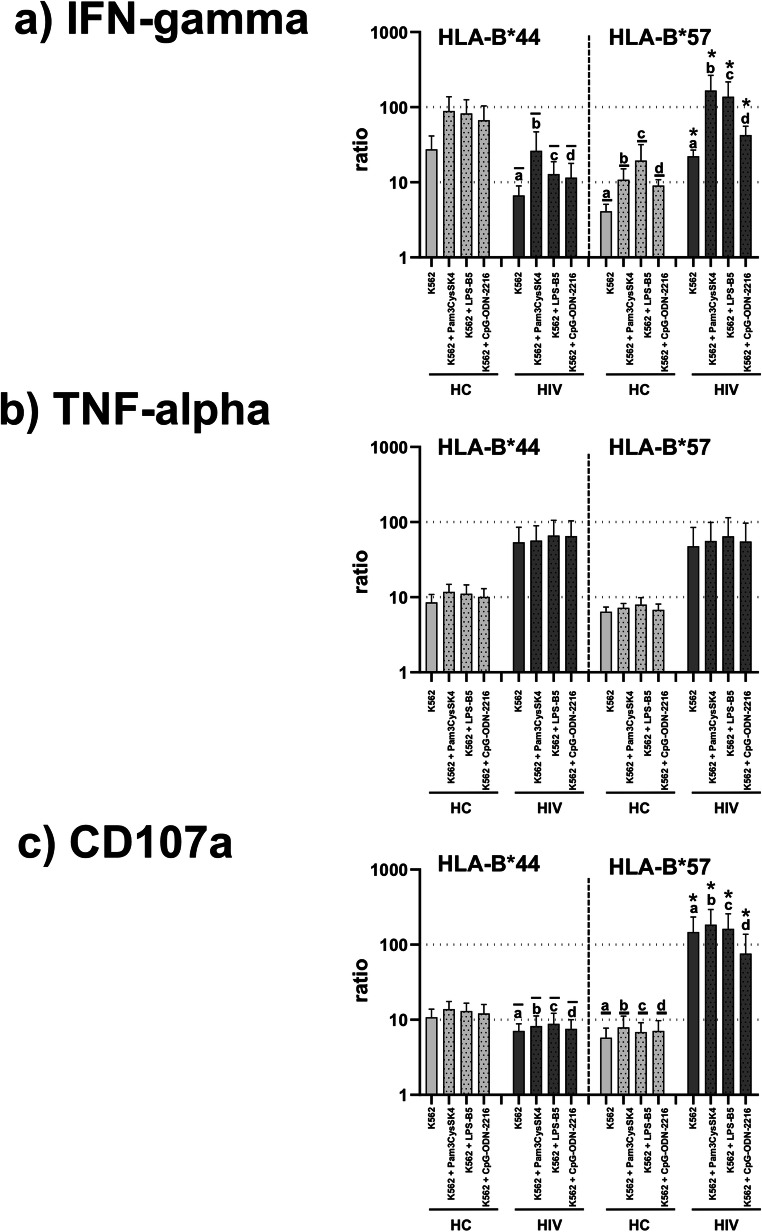
Response rates of IFN-gamma-, TNF-alpha- and CD107a-production in CD56^high^CD16^neg^ NK cells. This figure demonstrates enhanced response rates of IFN-gamma production (**a**) and degranulation (**c**) relative to baseline after stimulation with K562 cells in the CD56^high^CD16^neg^ NK cells of HLA-B*57-positive HIV patients compared to HIV patients with HLA-B*44 and HLA-B*57-positive healthy controls. In contrast to induction of TNF-alpha (**b**), response rates of IFN-gamma (**a**) and degranulation (**c**) were further enhanced in HLA-B*57-positive HIV patients compared to HIV patients with HLA-B*44 and HLA-B*57-positive healthy controls when CD56^high^CD16^neg^ NK cells were co-stimulated with K562 cells plus Pam3CysSK4, LPS-B5 and CpG-ODN-2216, respectively. *P* values refer to significances obtained by non-parametric Wilcoxon-matched pairs signed rank test and unpaired Mann-Whitney *U* test for the differences marked by letters. Error bars represent SEM and asterisks indicate significances *p* < 0.05 in pairwise statistical comparisons: In panels a and c, the letters “a*” refer to the comparisons between ratios of K562 stimulation in the HIV patients with HLA-B*57 versus ratios of K562-stimulation in HIV patients with HLA-B*44 ($\overline{a}$) and healthy controls with HLA-B*57 (a), respectively. The letters “b*”, “c*” and “d*” compare ratios after combined stimulation with K562 cells plus Pam3CysSK4 (b), LPS-B5 (c) and CpG-ODN-2216 (d) in HIV patients with HLA-B*57 versus HIV patients with HLA-B*44 ($\overline{b},\overline{c},\overline{d}$) and healthy controls with HLA-B*57 (b, c, d)

We analysed induction of IFN-gamma and TNF-alpha as well as degranulation in response to each TLR ligand, K562 cells and combined TLR ligands plus K562 cells separately in all NK cell subsets (CD56^dim^CD16^pos^, CD56^dim^CD16^neg^ and CD56^high^CD16^neg^). However, HLA-dependent changes were exclusively found in CD56^high^CD16^neg^ NK cells: in HLA-B*57-positive HIV patients, their frequencies of IFN-gamma-positive cells were significantly lower at baseline and after stimulation with K562 cells than in the HIV patients with HLA-B*44, whereas such differences were not observed concerning TNF-alpha and CD107a (Fig. 4). Of note, when analysing the IFN-gamma production and degranulation after stimulation with K562 cells as ratios relative to baseline production ratios in CD56^high^CD16^neg^ NK cells from HLA-B*57-positive HIV patients were conspicuously higher than in those from HLA-B*44-positive HIV patients and HLA-B*57-positive healthy controls, respectively (Fig. 5). After combined stimulation of NK cells with K562 cells plus either Pam3CysSK4, LPS-B5 or CpG-ODN-2216, the differences in the stimulated ratios between HLA-B*57- and HLA-B*44-positive HIV patients as well as HLA-B*57-positive healthy controls were further enhanced (Fig. 5).

## Discussion

Here, we report that bacterial TLR agonists induce increased amounts of IL-6/IL-1beta double-positive monocytes as well as IFN-gamma-positive NK cells with cytolytic activity in patients with virally suppressed HIV infection, which was substantially more pronounced in carriers of a HLA-B*57 type than in the HIV patients with HLA-B*44. It is noteworthy that these differences were particularly observed in the inflammatory cell subsets of the innate immune system, e.g. non-classical CD14^+^CD16^++^ monocytes and CD56^high^CD16^neg^ NK cells, both of which expand in HIV infection and remain activated even when viral replication is controlled by antiretroviral therapy [9]. Of note, similar differences between HLA-B*57- and HLA-B*44-positive cells were not observed in the HLA- and age-matched healthy controls. Differences between stimulation with Pam3CSK4 (TLR1/2 agonist), LPS-B5 (TLR4 agonist) versus CpG-ODN-2216 (TLR9 agonist) were noted indicating a specific effect. Furthermore, TNF-alpha induction was apparently not affected by HLA-B*57.

Our observations are in line with other reports which proposed that monocytes and NK cells rather than T cell activation contribute to morbidity and mortality of HIV patients on suppressive antiviral therapy: Jalbert and co-workers identified hyperinflammatory IL-1beta-enriched monocytes as a major source of IL-6 production and systemic inflammation in HIV-infected adults on antiretroviral therapy [10]. Here, our in vitro findings add the new information that TLR-triggered monocyte responses are modified by HLA-B*57 in patients with virally suppressed HIV infection. Of note, monocyte activation with increased release of IL-6 has repeatedly been reported to be associated with non-AIDS-defining events, immune aging and all-cause mortality in patients on antiretroviral therapy [11–15]. On the other hand, high IL-6 serum levels are a hallmark of imminent death in bacterial infection and sepsis [16, 17]. Thus, our in vitro findings seem to provide a hint why HLA-B*57 might be associated with an increased risk to die from bacterial infection in virally suppressed HIV patients [3].

NK cells coordinate responses to bacterial infections and also amplify antimicrobial functions of monocytes via IFN-gamma. Excessive NK cell activation and IFN-gamma production can over-activate the systemic inflammatory response during sepsis leading to physiological dysfunction and organ injury [18]. Although the role of NK cells is discussed controversially in virally suppressed HIV infection [9], enhanced cytotoxicity and IFN-gamma release of CD56^high^CD16^neg^ NK cells from HLA-B*57-positive HIV patients in response to TLR stimulation could contribute to mortality if such abundant NK cell activation also occurred in vivo. In addition, we studied monocytes in bulk preparations enabling cell–cell and cytokine interactions between monocytes and NK cells. Thus, it is possible that differential IFN-gamma release from NK cells between HLA-B*57-positive versus HLA-B*44-positive HIV patients might have contributed to enhance IL-6 and IL-1beta secretion in the monocytes. Since we did not observe any differences in the responses between HLA-B*57 versus HLA-B*44 of CD4^+^ or CD8^+^ T cells in our study, we consider interactions between monocytes and T cells to be a less likely factor. Nevertheless, we cannot prove at present which mechanisms underlie the observed differential responses between HLA-B*57-positive and HLA-B*44-positive cells. HIV infection and antiretroviral therapy are certainly important factors, since differential effects between HLA-B*57-positive and HLA-B*44-positive cells were not detected in the healthy controls.

In untreated HIV infection, HLA-B*57 delays disease progression due to exceptional efficacy of HLA-B*57-restricted T cells to recognise viral epitopes [1]. However, not all observations can be explained by differential interactions between HLA-B molecules and T cells. HLA-B*57 and HLA-B*44 encode isoleucine (Bw4-80Ile) and threonine (Bw4-80Thr) at position 80, also involved in their interactions with killer cell immunoglobulin–like receptors (KIRs) on NK cells. In particular, Bw4-80Ile can accelerate and delay HIV progression depending whether a patient carries the cognate receptor KIR3DS1, while Bw4-80Thr is neutral [19]. Bw80Ile/KIR3DS interactions generate multifunctional, TNF-alpha-positive NK cells [20], which, however, were not seen in our studies. On the other hand, long noncoding RNA *HCP5* is a polymorphic ancient insertion of an endogenous retrovirus next to the *HLA-B* locus involved in the regulation of innate and adaptive immunity. Its activity is altered by HIV infection via differential methylation patterns and its variants seem to differently affect HIV disease progression in close linkage to HLA*B57 types [21].

Concerning monocytes, HLA-B*57 and HLA-B*44 may act directly on cells from HIV patients via binding to leucocyte Ig–like receptors (LILRs) in a variable fashion. LILRs constitute a polymorphic alternative class of molecules expressed variably on multiple immune cells including myeloid cells. LILRs comprise activating and inhibitory receptors, which can bind classical (HLA-A, -B, -C) and non-classical (HLA-G) MHC class I molecules with differential affinity [22]. Thus, responses of immune cells are regulated by a balance between activating and inhibitory LILRs, putatively triggered differently by interactions with HLA-B*57 and HLA-B*44.

Owing to the great complexity of immune-regulatory elements and limitations to recruit sufficient numbers of HLA-typed HIV patients, we could not further dissect the causative cellular and molecular mechanisms. Nevertheless, our in vitro observations demonstrate that well-treated HIV infection HLA class I types can still modify secretion of certain pro-inflammatory monokines and NK cell activation triggered by PAMPs. These finding help to understand why HLA-B*57 in HIV infection may predispose patients to increased vulnerability for bacterial sepsis when viral replication is suppressed by antiretroviral drugs.

## Supplementary Information

Fig. S1 (JPEG 1.46 MB)

ESM 1 (TIFF 25.7 mb)

Fig. S2 (JPEG 1.61 MB)

ESM 2 (TIFF 25.7 mb)

## Data Availability

All data generated or analysed during this study are included in this published article and its supplementary information files.
