# Supplementary figures and images for: TLR agonists enhance responsiveness of inflammatory innate immune cells in HLA-B*57-positive HIV patients
Source: J Mol Med (Berl). 2020 Dec 5;99(1):147–58. doi: 10.1007/s00109-020-01996-7 (PMC7782382; doi:10.1007/s00109-020-01996-7)

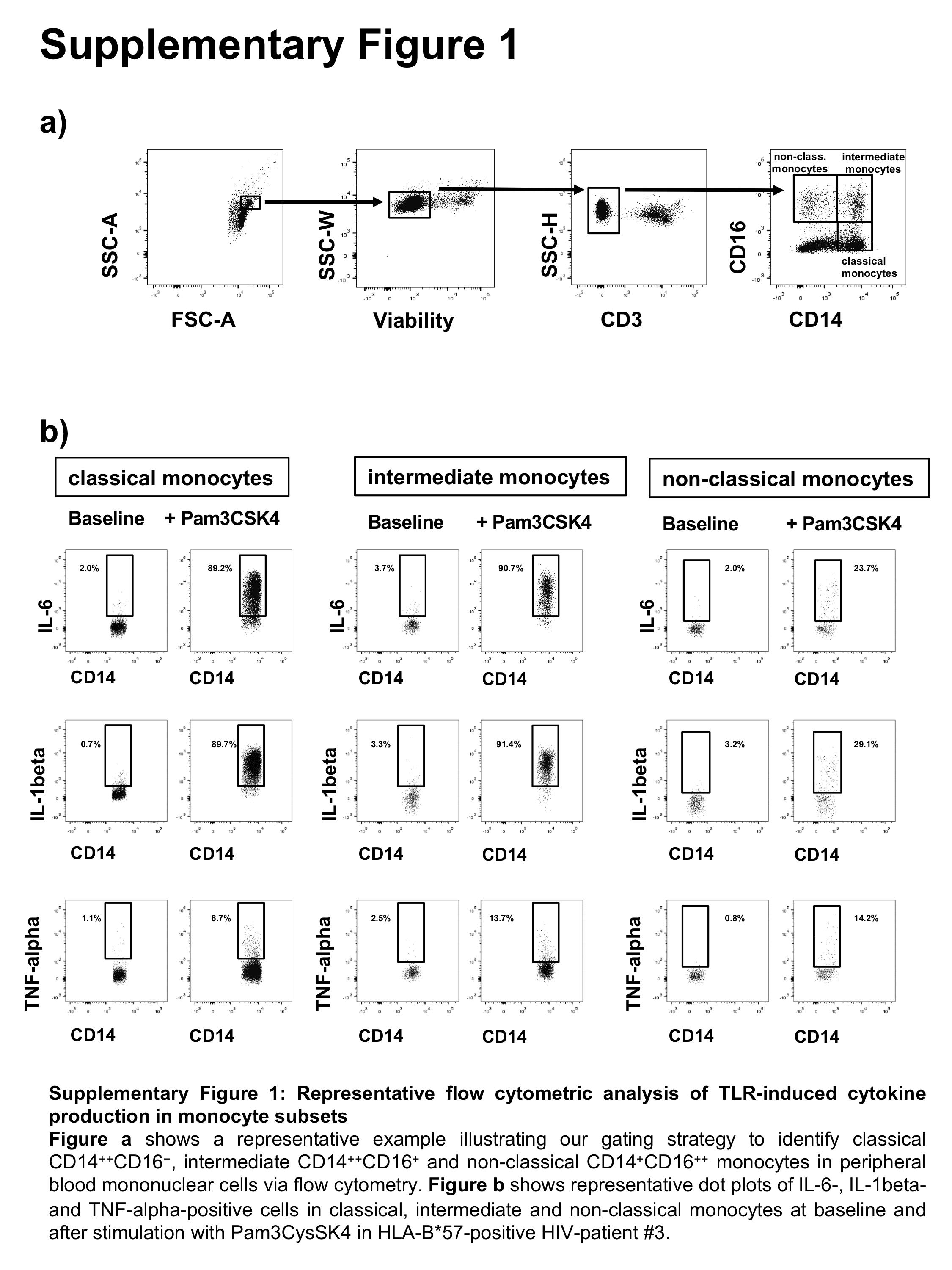

Supplement: Supplementary file 1 — (JPEG 1.46 MB) [file 109_2020_1996_Fig6_ESM.png]

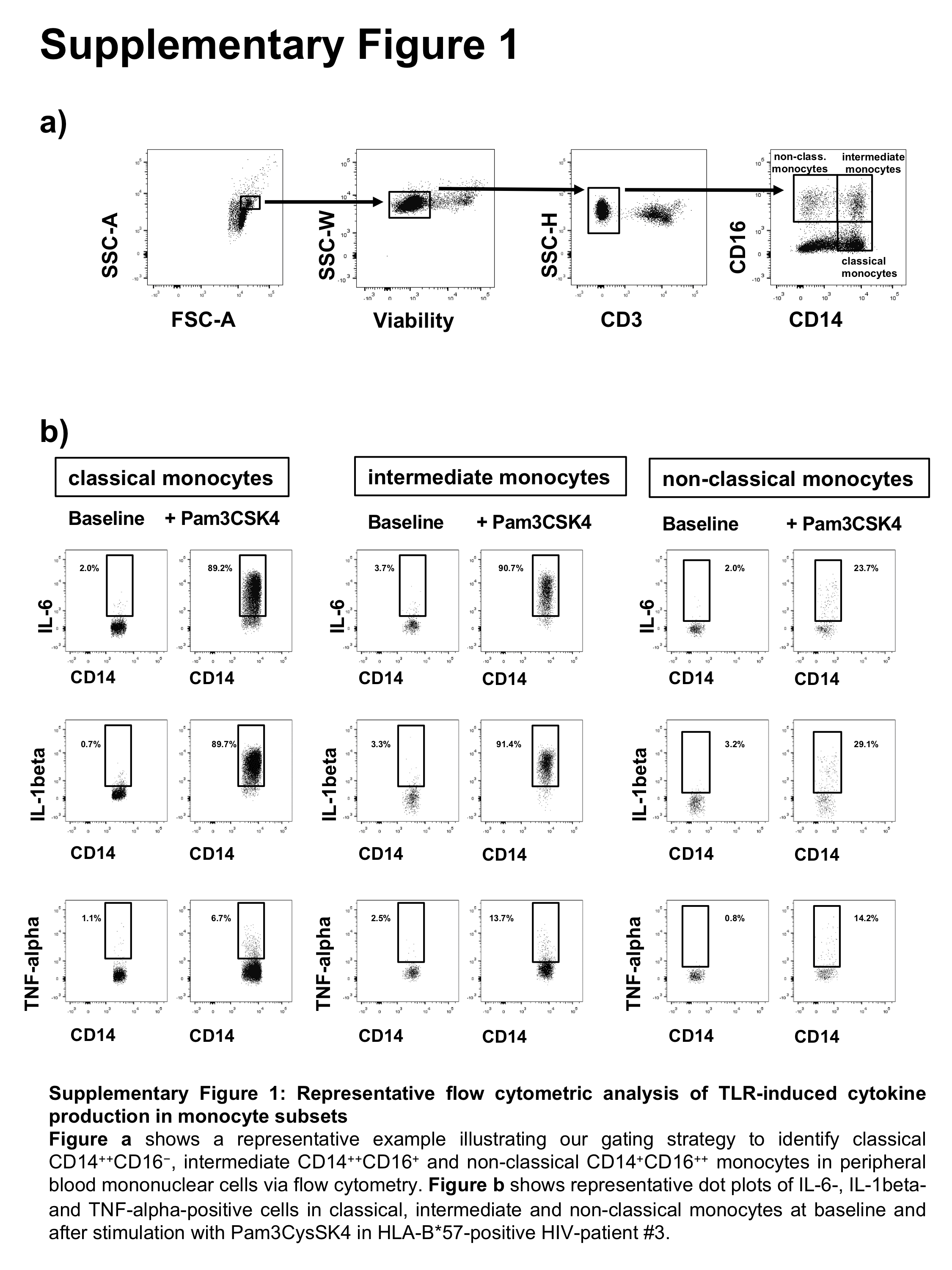

Supplement: Supplementary file 2 — (TIFF 25.7 mb) [file 109_2020_1996_MOESM1_ESM.tiff]

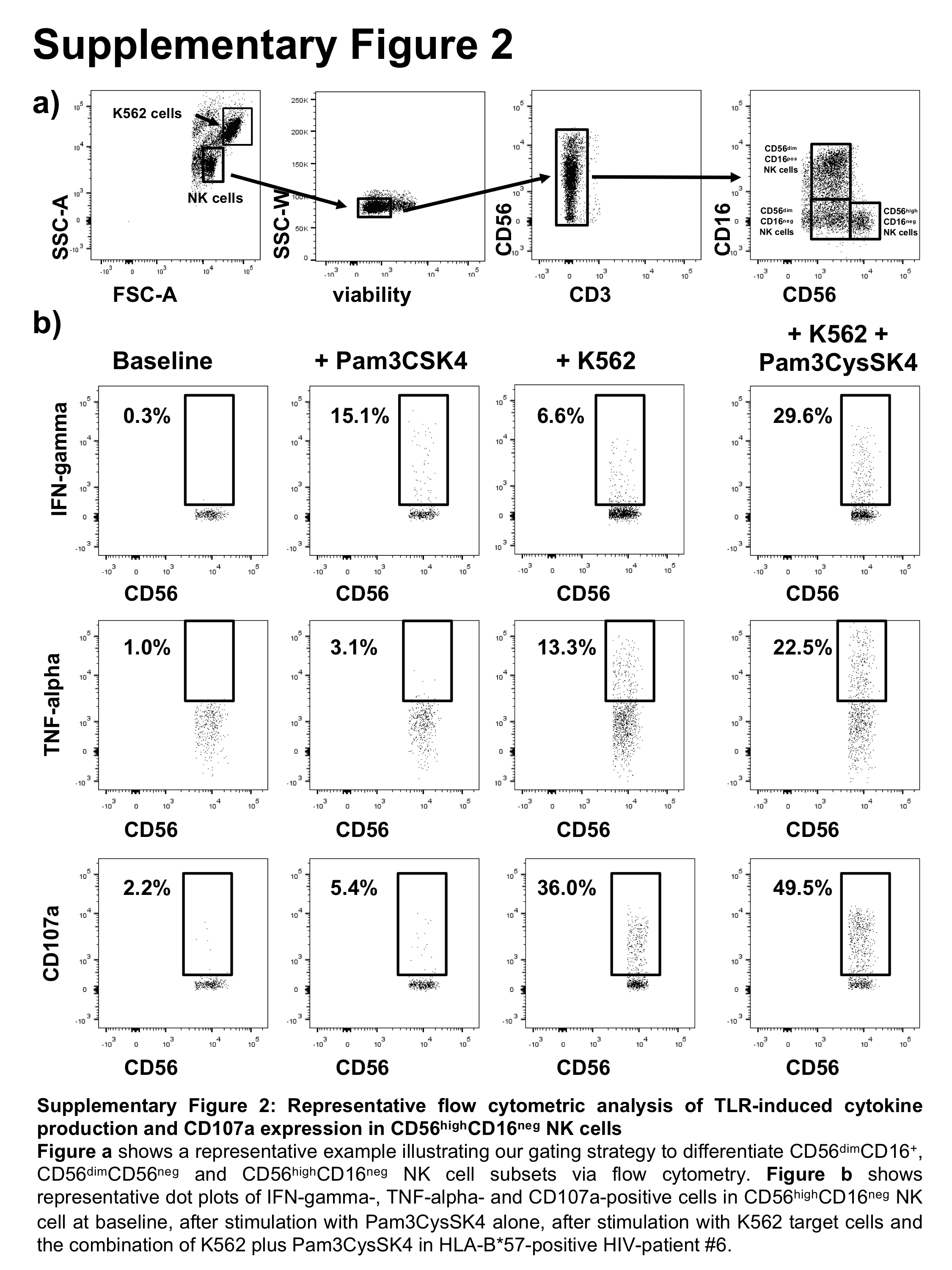

Supplement: Supplementary file 3 — (JPEG 1.61 MB) [file 109_2020_1996_Fig7_ESM.png]

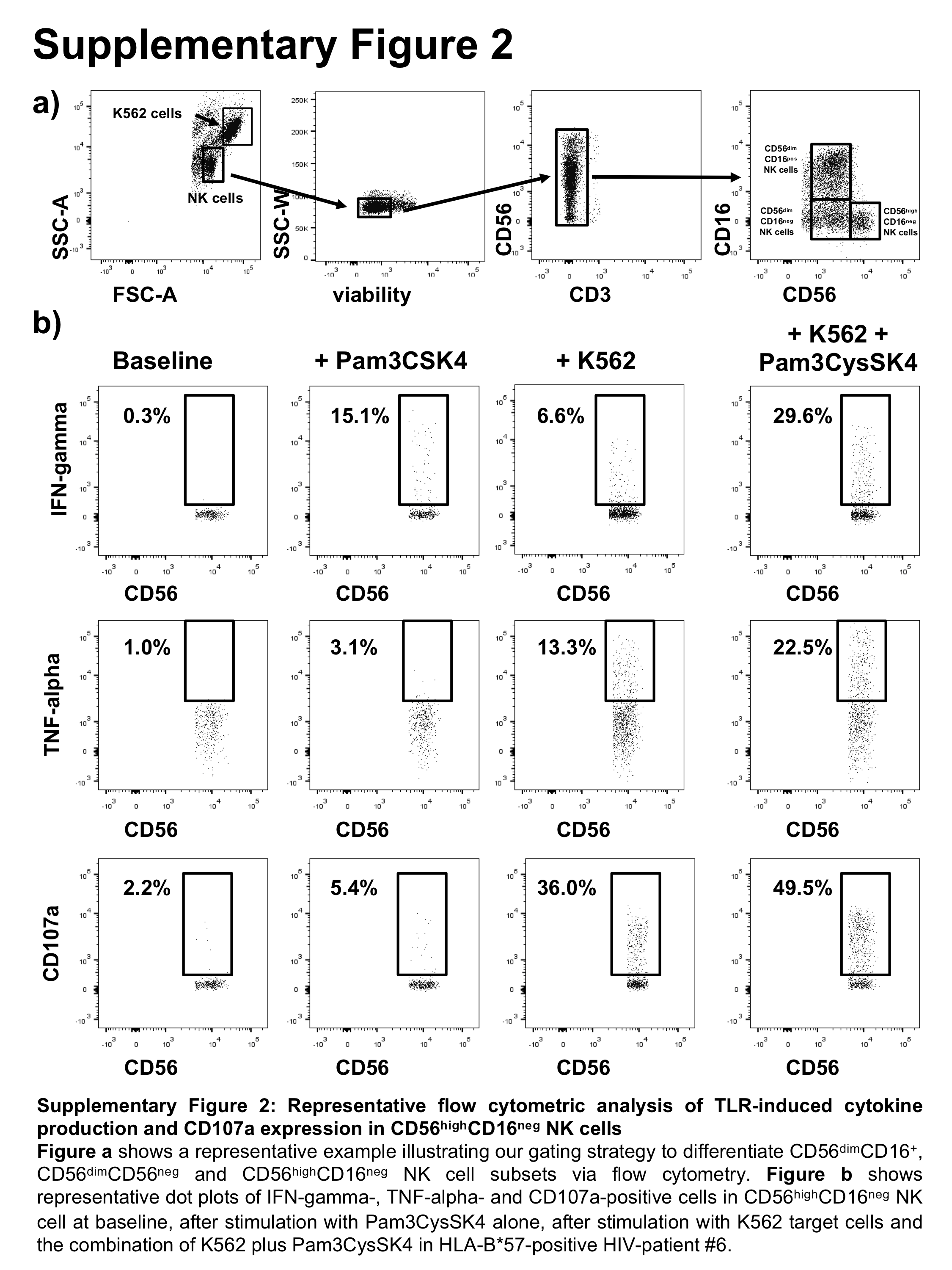

Supplement: Supplementary file 4 — (TIFF 25.7 mb) [file 109_2020_1996_MOESM2_ESM.tiff]
